# Reactivation of cocaine contextual memory engages mechanistic target of rapamycin/S6 kinase 1 signaling

**DOI:** 10.3389/fphar.2022.976932

**Published:** 2022-09-27

**Authors:** Xiangdang Shi, Eva von Weltin, Emma Fitzsimmons, Chau Do, Carolina Caban Rivera, Chongguang Chen, Lee-Yuan Liu-Chen, Ellen M. Unterwald

**Affiliations:** Center for Substance Abuse Research and Department of Neural Sciences, Lewis Katz School of Medicine at Temple University, Philadelphia, PA, United States

**Keywords:** conditioned place preference, rapamycin, mechanistic target of rapamycin complex 1, reconsolidation, cocaine, p70S6 kinase, Arc/Arg3.1

## Abstract

Mechanistic target of rapamycin (mTOR) C1 and its downstream effectors have been implicated in synaptic plasticity and memory. Our prior work demonstrated that reactivation of cocaine memory engages a signaling pathway consisting of Akt, glycogen synthase kinase-3β (GSK3β), and mTORC1. The present study sought to identify other components of mTORC1 signaling involved in the reconsolidation of cocaine contextual memory, including eukaryotic translation initiation factor 4E (eIF4E)-eIF4G interactions, p70 S6 kinase polypeptide 1 (p70S6K, S6K1) activity, and activity-regulated cytoskeleton (*Arc*) expression. Cocaine contextual memory was established in adult CD-1 mice using conditioned place preference. After cocaine place preference was established, mice were briefly re-exposed to the cocaine-paired context to reactivate the cocaine memory and brains examined. Western blot analysis showed that phosphorylation of the mTORC1 target, p70S6K, in nucleus accumbens and hippocampus was enhanced 60 min following reactivation of cocaine memories. Inhibition of mTORC1 with systemic administration of rapamycin or inhibition of p70S6K with systemic PF-4708671 after reactivation of cocaine contextual memory abolished the established cocaine place preference. Immunoprecipitation assays showed that reactivation of cocaine memory did not affect eIF4E–eIF4G interactions in nucleus accumbens or hippocampus. Levels of *Arc* mRNA were significantly elevated 60 and 120 min after cocaine memory reactivation and returned to baseline 24 h later. These findings demonstrate that mTORC1 and p70S6K are required for reconsolidation of cocaine contextual memory.

## Introduction

Drug reward memories are reactivated by exposure to cues that were previously associated with drug use, and then undergo a process of reconsolidation through synaptic plasticity which strengthens the memory (Mactutus et al., 1979; Przybyslawski and Sara, 1997). Reactivated memories become unstable and can be modified through pharmacological or behavioral manipulations that disrupt the reconsolidation process (Nader et al., 2000; Walsh et al., 2018). Since drug-associated cues can trigger craving and relapse, pharmacological interference with reconsolidation-related plasticity may be a useful approach to prevent relapse (Saladin et al., 2013). Understanding the molecular processes involved in reconsolidation of cocaine memories is needed to develop therapeutic strategies to help dampen cue-induced craving and relapse. Previous reports have demonstrated that cocaine engages molecular signaling pathways that are involved in associative learning processes, such as MEK/ERK/CREB/Elk-1 signaling pathway (Miller and Marshall, 2005), and NMDA/PP1/GSK3/mTORC1 signaling pathway (Shi et al., 2014; Shi et al., 2019).

Reconsolidation is a process that requires *de novo* protein synthesis after memory reactivation (Nader et al., 2000). More recently, mechanistic (previously mammalian) target of rapamycin (mTOR) has gained much attention for its role in regulating protein synthesis during memory reconsolidation (Roesler, 2017; Jarome et al., 2018; Radiske et al., 2021). mTOR is a serine-threonine protein kinase that forms two distinct multiprotein complexes, mTORC1 and mTORC2 (Loewith et al., 2002). mTOR interacts with the adaptor protein Raptor and forms a complex as mTOR complex 1 (mTORC1). mTORC1 regulates protein translation by controlling the phosphorylation state of its two main downstream substrates, eukaryotic translation initiation factor 4E (eIF4E)-binding proteins (4EBPs) and ribosomal S6 kinases (S6Ks) (Raught et al., 2001). Hypophosphorylated 4E-BPs bind strongly to eIF4E, whereas mTORC1-induced phosphorylation of 4E-BP1 results in its release from eIF4E, which in turn binds to eIF4G, allowing the formation of eIF4F complex to promote the translation initiation (Gingras et al., 2001). mTORC1-induced phosphorylation of S6K1 regulates translation initiation and elongation through phosphorylation of several downstream effectors, including eIF4B (Raught et al., 2004) and eukaryotic elongation factor 2 (eEF2) kinase (Browne and Proud, 2002).

Evidence suggests that mTORC1 signaling pathway plays an important role in memory reconsolidation. For example, both consolidation and reconsolidation of fear memories are dependent on mTORC1 activity and can be disrupted by inhibition of mTORC1 with rapamycin (Blundell et al., 2008; Gafford et al., 2011; Mac Callum et al., 2014). Moreover, the persistence of fear memory requires both eIF4E-eIF4G interaction and S6K1 activation. mTORC1 plays an essential role in reconsolidation of drug-related memories as shown in studies using conditioned place preference (Lin et al., 2014) or self-administration models (Barak et al., 2013; Zhang et al., 2021). However, whether eIF4E–eIF4G interactions and S6K1, two mTORC1 downstream targets, are involved in reconsolidation of cocaine contextual memories remains unknown.

As an effector immediate-early gene, the activity-regulated cytoskeletal-associated protein (Arc/Arg3.1) is necessary for long-term memory formation (Guzowski et al., 2000). Arc has been implicated in synaptic plasticity (Plath et al., 2006; Rial Verde et al., 2006), memory consolidation (Plath et al., 2006; Ploski et al., 2008), and reconsolidation of Pavlovian fear memory (Maddox and Schafe, 2011; Chia and Otto, 2013). Arc is regulated by drug-related memories including alcohol (Barak et al., 2013), cocaine (Hearing et al., 2008a; Hearing et al., 2008b; Alaghband et al., 2014), and morphine (Lv et al., 2015). The expression of *Arc* mRNA can be used as a marker for neuronal plasticity. Unlike the mRNAs of other immediate early genes that remain in the cell body, newly synthesized *Arc* mRNA is rapidly distributed throughout the dendritic segments near active synapses after neuronal activation (Steward et al., 2014).

The current study sought to determine the role of mTORC1 and its downstream effectors in the reconsolidation of cocaine contextual memories. A mouse model of cocaine place conditioning was used to establish cocaine contextual memories. The regulation of phosphorylated p70S6K, a readout of mTORC1 activity, was examined following reactivation of cocaine memory and demonstrated an activation of mTORC1 60 min after memory reactivation. Further experiments examined the functional role of mTORC1 and p70S6K in the process of reconsolidation of cocaine-associated memory. Regulation of targets of mTORC1 in the nucleus accumbens and hippocampus were investigated including the eIF4E–eIF4G complex and *Arc* mRNA expression. The study focused on the nucleus accumbens and hippocampus because little is known about the potential role of mTORC1 signaling in these regions as related to the reconsolidation of cocaine reward memories. Neurons in both the nucleus accumbens and hippocampus are activated during reactivation of cocaine-associated memory (Soderman and Unterwald, 2008; Shi et al., 2014) and evidence suggests that the nucleus accumbens (Miller and Marshall 2005; Milekic et al., 2006; Théberge et al., 2010) and hippocampus (Milekic et al., 2006; Sakurai et al., 2007) may participate in the reconsolidation of drug-associated memory. The nucleus accumbens receives convergent glutamatergic projections from ventral hippocampus, medial prefrontal cortex and basolateral amygdala (Phillipson and Griffiths, 1985; Sesack and Grace, 2010; Britt et al., 2012; Russo and Nestler, 2013) which encode stimuli such as the context and cues that predict rewarding events (Everitt and Wolf, 2002; Kelley, 2004; Pennartz et al., 2011; Floresco, 2015). Information transmitted from the basolateral amygdala or the hippocampus in response to memory consolidation and reconsolidation is further modulated within the accumbens (Kerfoot and Williams, 2018; Torregrossa et al., 2019). Thus, nucleus accumbens may function as a coordination hub for the reconsolidation of cue-drug memory (Exton-McGuinness and Milton, 2018).

## Materials and methods

### Animals

Male CD-1 mice (8 weeks old on delivery, Charles River Laboratories, Wilmington, MA) were housed in groups of four per cage under a 12-h light/dark cycle (7:00 a.m./7:00 p.m.) without additional enrichment objects. Mice had access to standard chow and water *ad libitum*. Animals were housed for 5 days before experiments began and were weighed daily. Behavioral procedures were conducted between 13:00–17:00. Animal procedures were performed in compliance with the National Institutes of Health guidelines for the Care and Use of Laboratory Animals, and animal use was reviewed and approved by Temple University Institutional Animal Care and Use Committee.

### Drugs

Cocaine hydrochloride was generously supplied by the National Institute on Drug Abuse Drug Supply Program, dissolved in sterile saline, and injected intraperitoneally (i.p.) in a volume of 3 ml/kg body weight. An equal volume of saline served as the vehicle control for cocaine. Rampaycin was purchased from LC labs (Woburn, MA), and prepared in 5%DMSO/5%Tween80/saline. PF4708671 was purchased from Selleck Chemicals (Houston, TX), and prepared in 30%PEG-400/0.5%Tween-80/5%propylene glycol/saline. Both rapamycin and PF4708671 were injected i. p. in the volume of 10 ml/kg body weight. Equal volumes of 5%DMSO/5%Tween80/saline and 30%PEG-400/0.5%Tween-80/5%propylene glycol/saline served as the vehicle controls for rapamycin and PF4708761, respectively.

### Cocaine-induced conditioned place preference and reactivation of cocaine contextual memory

The procedures were the same as described in our prior publication (Shi et al., 2019). Place conditioning occurred in rectangular plastic chambers (45 × 20 × 20 cm) consisting of two unique compartments, one with white and black vertical striped walls and smooth flooring and the other with white walls with black circles and rough flooring. Illumination in both compartments was equal. The two compartments were separated by a removable wall during conditioning and a door during testing. Prior studies showed that >90% of mice tested in these chambers show no initial preference to either compartment. An unbiased conditioned place preference (CPP) procedure was used wherein the mice were randomly assigned to receive cocaine in one or the other compartment in a counterbalanced design without a pre-test. Mice were injected with 10 mg/kg ip cocaine or saline and were immediately confined to one compartment of the conditioning chamber where they remained for 30 min. Conditioning occurred once per day for eight consecutive days resulting in four conditioning sessions with saline in one compartment and four sessions with cocaine in the opposite compartment of the conditioning chamber. The test for place preference occurred on day 9, when mice had access to both compartments for 15 or 30 min in a drug-free state. The time spent in each compartment was recorded. Preference scores were calculated as: [time spent in the cocaine-paired compartment] minus [time spent in the saline-paired compartment] and reported in seconds. Mice that did not show a cocaine place preference on day 9 were eliminated from further study. On day 10, 24 h following assessment of cocaine place preference, one group of mice was re-exposed to the previously cocaine-paired compartment for 10 min to reactivate cocaine-associated memory, whereas another group was kept in the home cage and made up the no exposure control group.

### Brain tissue collection

Brains were obtained 30 min, 60 min, 120 min, or 24 h following memory reactivation; mice were briefly exposed to CO_2_ anesthesia followed by decapitation. In experiment 1, the nucleus accumbens and hippocampus were dissected, frozen on dry ice and store at −80°C. In experiment 4-5, brains were removed, flash frozen in isopentane (−40°C) and stored at −80°C. One hemisphere of each frozen brain was used for RNA extraction. In this case, brains were sectioned on a cryostat microtome and 1 mm punches were used to obtain the nucleus accumbens, dorsal hippocampus, and ventral hippocampus (Paxinos and Watson, 2007) from four slices 200 µm thick. Tissue punches were placed in RNAlater-ICE (Invitrogen, Waltham, MA), incubated overnight at −20°C before storage at −80°C. The other hemispheres were used for immunoprecipitation assays. In this case, the nucleus accumbens, dorsal hippocampus, and ventral hippocampus were dissected on ice using a mouse brain matrix, frozen on dry ice, and store at −80°C.

### Quantitative reverse transcriptase polymerase chain reaction

Total RNA was extracted using a Quick-RNA Miniprep kit (Zymo Research, Irvine, CA), and RNA concentration was measured using a NanoDrop 2000 spectrophotometer. RNA samples were diluted to the same RNA concentration before cDNA was synthesized using the High-Capacity cDNA Reverse Transcription Kit (Applied Biosystems, Waltham, MA). Quantitative RT-PCR (RT-qPCR) was performed using TaqMan Fast Advanced Master Mix and TaqMan Gene Expression Assays for *Arc* (Mm00479619_g1), and the control 18S rRNA (Hs99999901_s1). Relative fold change in gene expression level was calculated using the 2^–∆∆Ct^ method (Livak and Schmittgen, 2001).

### Immunoprecipitation assay

Tissue was sonicated in ice cold lysis immunoprecipitation buffer containing in 50 mM Tris (pH7.4), 0.15 M NaCl, 1% NP-40 (IGEPAL CA-630, Sigma), 0.5% DOC (sodium deoxycholate), Complete Mini protease inhibitor tablet (Pierce), and Halt™ phosphatase inhibitor cocktail (Thermo Fisher Scientific). Nucleus accumbens and hippocampus were homogenized in 250 ul of lysis buffer to fine suspension and centrifuged at 12,000 g for 10 min at 4°C. The supernatant was transferred to a new tube, protein concentration was measured by means of the BCA assay (Pierce). Homogenates (125–250 ug) were precleared by adding 40 ul Protein A/G PLUS agarose (SC-2003, Santa Cruz) and incubated for 30 min at 4°C. The supernatant was saved and incubated with elF4G antibody conjugated to Protein A/G Plus-Agarose (20 ug/10ul, SC-133155 AC, Santa Cruz) overnight at 4°C on the rocker. The agarose-antibody-antigen complex was collected by centrifugation (12,000 g for 20 s) at 4°C. Immunoprecipitated complexes were washed three times in cold lysis buffer and once in cold wash buffer (0.1% NP-40, 25 mM Tris (pH7.4), 1 mM EDTA). After the final wash, the immunoprecipitated complexes were eluted with gel loading buffer (4% SDS, 25 mM Tris (pH7.4)). The resulting elutes were saved for western blot analysis.

### Western blot analysis

For experiment 1, tissue was homogenized in ice cold lysis buffer and supernatant was collected by centrifugation as mentioned above. Samples were diluted in gel loading buffer and boiled for 5 min, 10 µg of protein was separated on 4–15% Tris–HCl Bio-Rad Ready-gels (Cat#: 4561026. Bio-Rad Laboratories, Hercules, CA) and transferred to nitrocellulose membranes. Membranes were blocked in Odyssey blocking buffer (LI-COR Biosciences, Lincoln, NE) and incubated overnight at 4°C with the following primary antibodies: anti-phospho-p70S6K (1:1,000, Cat#9205, Cell Signaling, Beverly, MA) and anti-p70S6K (1:1,000; Cat#9202, Cell Signaling). Membranes were washed in TTBS and incubated with anti-rabbit (1:15,000, Cat# 926–32211) or anti-mouse (1:15, 000, Cat# 926–68070) secondary antibodies conjugated to two different infra-red dyes (LI-COR Biosciences, Lincoln, NE). Bands were visualized using the Odyssey infrared imaging system and software (Li-COR), and intensities were quantitated using ImageJ software. Levels of phospho-p70S6K were expressed as a ratio to total P70SK6 for each sample. The mean ratio of the no exposure control group was set to 100% and all data points are presented relative to that value.

To detect the efficiency of the immunoprecipitation for experiments 4–5, five µg of input (total lysate) and 50% or 100% of eluates were loaded to 4–15% Tris-glycine gradient gels (Bio-Rad). Separated proteins were transferred onto nitrocellulose membranes. Membranes were blocked with 3% BSA in Tween-TBS for 1 h and incubated overnight at 4°C in the following primary antibodies; anti-eIF4G1 (1:1,500, cell signaling, #2498), anti-eIF4E (1:1,500, #9742), followed by secondary antibody (HRP-conjugated goat anti-rabbit IgG, 1:10,000, Jackson Immuno Research, 211–032–171) incubation for 1 h at room temperature. Membranes were washed and proteins were detected by enhanced chemiluminescence reagent (ECL+; GE Healthcare). Membranes were stripped and incubated with anti-GAPDH antibody (1:1,500, Cat# Cell Signaling, Beverly, MA) to normalize the optical density values of eIF4G or eIF4E (input). Images were visualized by FUJIFILM LAS-1000 imaging system and staining intensities of bands were quantitated using the ImageJ software. Band density values were normalized to GAPDH (input eIF4G or eIF4E), or eIF4G (immunoprecipitation eIF4E: eIF4G).

### Experimental design

Experiment 1 Regulation of mTORC1 activity following reactivation of cocaine contextual memories.

**FIGURE 1:**
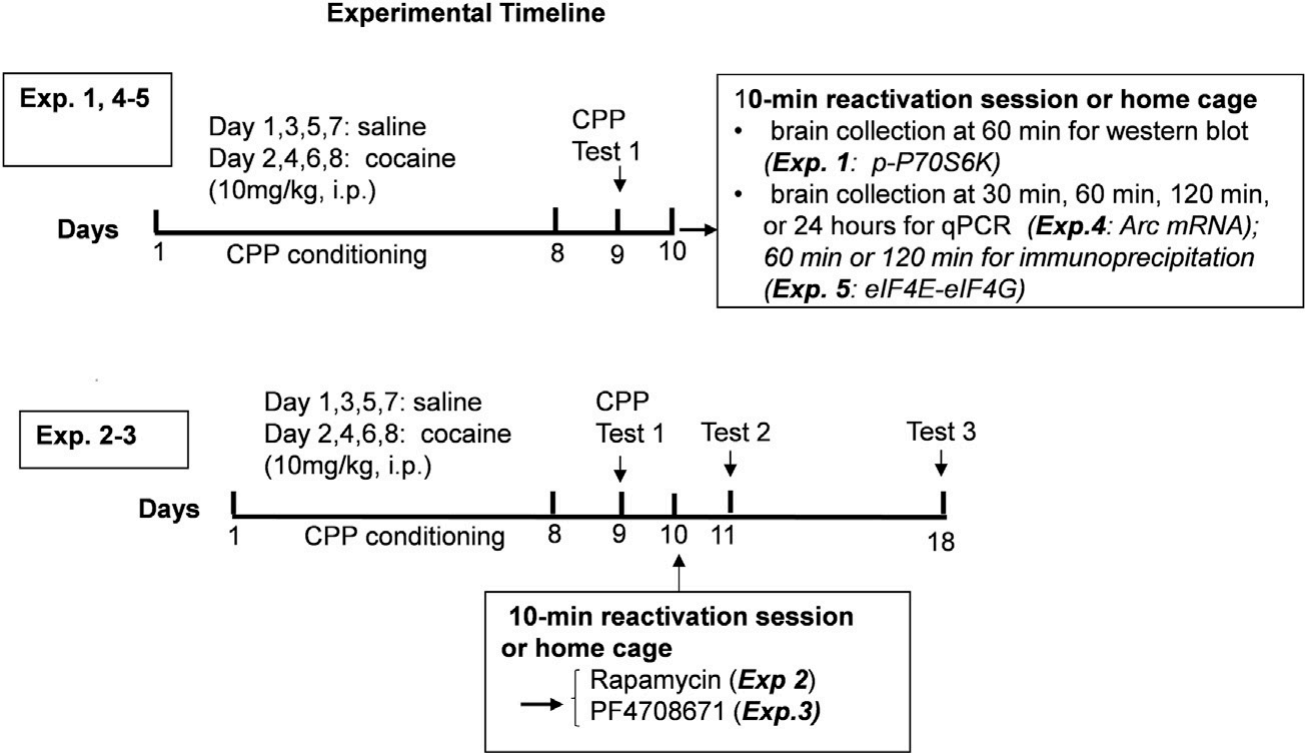
Experimental timeline.

To assess the regulation of mTORC1 activity after cocaine memory reactivation, 20 mice underwent cocaine conditioned place preference See Figure 1. Cocaine CPP was established in 15 out of the 20 mice; five mice that had a CPP score of <180 s (in a 30 min test session) were removed from further study. 24 h following the test for cocaine place preference, half the mice were re-exposed to the previously cocaine-paired compartment for 10 min to reactivate cocaine-contextual memory, while the others remained in the home cage and served as no exposure controls. Brains were collected 60 min following the 10-min reactivation session. Nucleus accumbens and hippocampus were dissected, stored at -80°C, and prepared for measurement of mTORC1 activity by immmunoblotting as described above.

Experiment 2 Effect of mTORC1 inhibition on reconsolidation of cocaine contextual memories.

Five groups of mice underwent cocaine place conditioning and were tested on day 9 for preference; 47 out of 60 mice had CPP scores >180 s and continued in the study. On day 10, three groups of mice were re-exposed to the compartment previously paired with cocaine for 10 min followed by administration of the mTORC1 inhibitor rapamycin (0, 1, and 5 mg/kg, i. p.). The other two groups served as no-reactivation controls, remained in their home cages and were injected with vehicle or rapamycin (5 mg/kg, i. p.) according to the same time schedule. On days 11 and 18, all mice were re-tested for place preference without further drug injections or conditioning sessions.

Experiment 3 Effect of p70S6K inhibition on reconsolidation of cocaine contextual memories.

To investigate the role of p70S6 kinase, an effector downstream of mTORC1 in the reconsolidation of cocaine contextual memories, 52 mice underwent cocaine conditioned place preference. On test day 9, 41 mice showed a cocaine place preference and were used in the subsequent memory test. The procedures were the same as described in Experiment 2 except that mice were injected with the p70S6K inhibitor PF-4708671 (0, 10, 50 mg/kg ip) on day 10.

Experiment 4 Regulation of *Arc* mRNA expression following reactivation of cocaine contextual memories.

To assess the effects of reactivation of cocaine-associated memories on *Arc* expression, 96 mice underwent cocaine conditioned place preference; 24 mice were excluded for failure to reach the criteria of >90 s CPP score during a 15 min test session for place preference on day 9. Mice that showed a cocaine place preference were re-exposed to the cocaine context or remained in the home cage on day 10. Brains were collected 30 min, 60 min, 120 min, or 24 h following the 10-min reactivation session. Nucleus accumbens and hippocampus (dorsal and ventral) samples were prepared for *Arc* mRNA analysis as described above.

Experiment 5 eIF4E-eIF4G interactions after reactivation of cocaine reward memory.

To investigate effects of reactivation of cocaine associated memories on eIF4E-eIF4G interactions, 48 mice underwent cocaine conditioned place preference; 36 mice showed a cocaine place preference on day 9 and continued in the study. Mice were re-exposed to the cocaine context or remained in the home cage on day 10. Brains were collected 60 or 120 min following the 10-min reactivation session. and the nucleus accumbens and hippocampus were prepared for IP and protein analysis as described above.

### Data analysis

Conditioned place preference data were analyzed using repeated measures two-way ANOVA (treatment × test day in experiment 2 and 3). Levels of p-p70S6K (Exp. 1), Arc mRNA (Exp. 4), and eIF4E bound to eIF4G (Exp. 5) were analyzed using two-tailed t-test. Statistical analyses were performed using GraphPad Prism 9 (La Jolla, CA).

## Results

### Experiment 1: Reactivation of cocaine contextual memories activates mechanistic target of rapamycin complex 1

**FIGURE 2:**
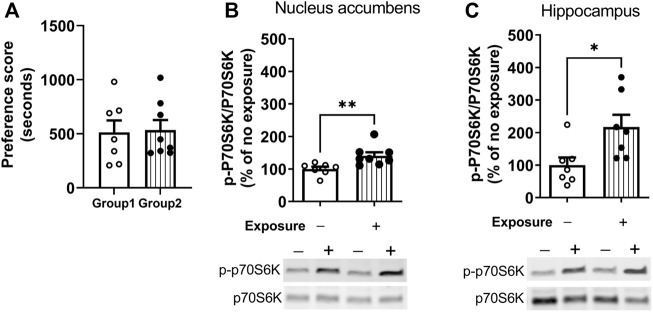
Regulation of mTORC1 activity in the nucleus accumbens and hippocampus after reactivation of cocaine memories. Place conditioning with cocaine 10 mg/kg occurred once daily for 8 days **(A)** Cocaine place preference scores from day 9 are shown; similar preference for the cocaine-paired compartment was found for two groups of mice. On day 10, mice were re-exposed to the cocaine compartment for 10 min to reactivate cocaine contextual memories (exposure group) or left in home cage (no exposure control group), and brains harvested 60 min later. Levels of phospho-p70S6K (p-p70SK6) and total p70S6K were quantified by Western blot and expressed as a ratio of p-p70S6K/p70S6K for each sample. p-p70S6K was significant induced 60 min following reactivation of cocaine memories in nucleus accumbens **(B)** and hippocampus **(C)**, as shown by comparison of the exposure group with no exposure controls. Mean ratio of p-p70S6K/p70S6K for the no exposure controls is set to 100% and data points are expressed relative to 100% of no exposure controls. *p < 0.05, **p < 0.01 exposure vs. no exposure. Data are expressed as means ± SEM. N = 7–8/group.

The measurement of phosphorylation of the mTORC1 target, p70S6K, was used to assess the time-course of regulation of mTORC1 activity after cocaine memory reactivation (Hay and Sonenberg, 2004). Cocaine place preference was determined on day 9 following 8 days of conditioning. Two groups of mice showed similar preference for the cocaine-paired context (Figure 2A). On day 10, one group of mice was placed back into the previously paired cocaine compartment for 10 min while the others remained in home cages. Brains were obtained 60 min later. Representative immunoblots of nucleus accumbens (Figure 2B) and hippocampus (Figure 2C) from mice with (Exposure +) or without exposure (Exposure -) to the cocaine context are presented. (full-length blots are included in the Supplementary Figure S1,S2). The level of p-p70S6K was significantly higher 60 min post reactivation as compared with no exposure controls in the nucleus accumbens (t = 3.043, df = 13, *p* < 0.01). A similar pattern of p-p70S6K regulation was found in the hippocampus (Figure 2C); levels of p-p70S6K were significantly higher after memory reactivation as compared with no exposure controls (t = 2.643, df = 12, *p* < 0.05). These results demonstrate an increase in mTORC1 activity in the nucleus accumbens and hippocampus following cocaine contextual memory reactivation.

### Experiment 2: Inhibition of mTORC1 with rapamycin disrupted the reconsolidation of cocaine contextual memories

**FIGURE 3:**
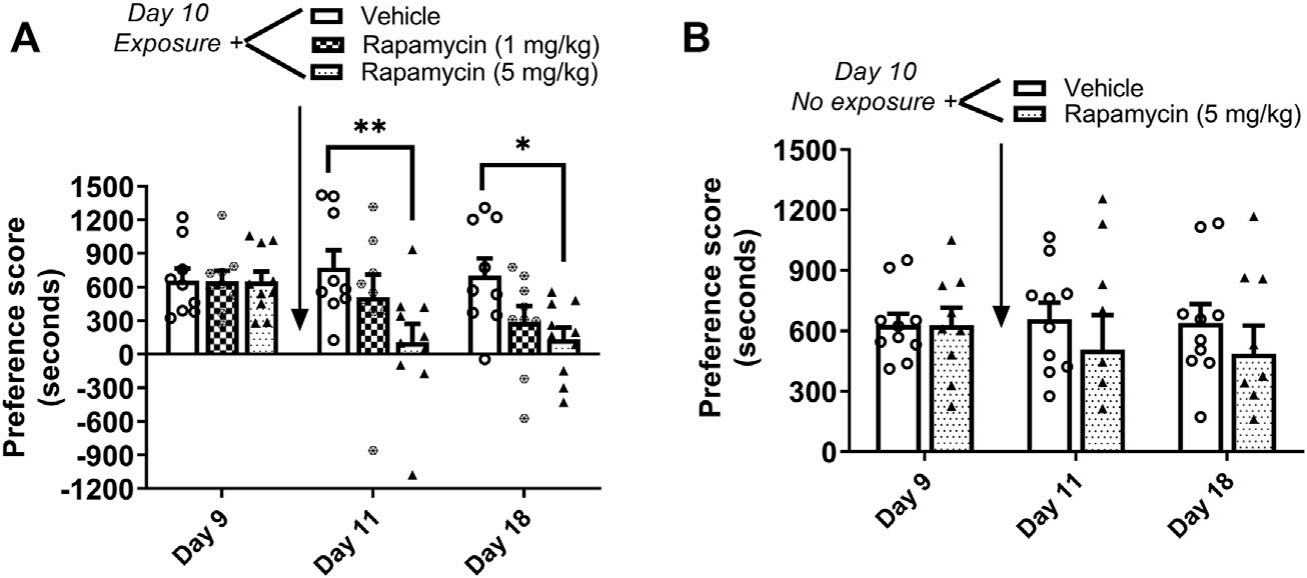
Rapamycin disrupts reconsolidation of cocaine memories. **(A)** Condition place preference was established with cocaine 10 mg/kg in three groups of mice as shown on day 9 during a 30-min post-conditioning test session. On day 10, mice were re-exposed to the cocaine-paired context in a drug-free state for 10 min to reactivate cocaine-associated memories. Rapamycin (1 or 5 mg/kg ip) or vehicle was administered immediately after exposure to the cocaine compartment. Mice were re-tested for cocaine place preference 24 h and 7 days later. Rapamycin (5 mg/kg) significantly abolished the previously established place preference when tested 24 h and 7 d after administration (days 11 and 18). Mice injected with vehicle maintained a significant cocaine place preference on days 11 and 18. **(B)** Condition place preference was established with cocaine 10 mg/kg in two groups of mice as shown on day 9. On day 10, mice remained in their home cages in the testing room and were injected with rapamycin (5 mg/kg ip) or vehicle. Mice were re-tested for cocaine place preference 24 h and 7 days later. Rapamycin administered in the home cage (ie, no exposure to the cocaine context) did affect the previously established cocaine place preference; both vehicle and rapamycin groups maintained a cocaine place preference when retested on days 11 and 18. *p < 0.05, **p < 0.01 vs. vehicle, N = 9/group. Data are expressed as means ± SEM.

Mice underwent 8 days of cocaine place conditioning followed by a test for preference on day 9. Mice were re-exposed to the cocaine-paired context on day 10 to reactivate cocaine memories, followed immediately by administration of the mTORC1 inhibitor rapamycin (1 or 5 mg/kg ip) or vehicle. Results demonstrate that rapamycin dose dependently reduced the previously established cocaine place preference when retested on days 11 and 18, as shown in Figure 3A. Repeated measures two-way ANOVA of preference scores revealed a significant interaction (F_4,50_ = 2.66, *p* < 0.05) between rapamycin treatment (F_2,50_ = 4.12, *p* < 0.05) and test day (F_2,50_ = 4.6, *p* < 0.05). Post hoc tests indicated that administration rapamycin (5 mg/kg) immediately following reactivation of cocaine reward memories significantly attenuated preference for the cocaine-paired chamber when tested 24 h later (*p* < 0.01 vs. vehicle, day 11) or 7 days later (*p* < 0.05 vs. vehicle, day 18). In order to demonstrate that rapamycin was interfering with memory reconsolidation, a control experiment was performed wherein vehicle or rapamycin (5 mg/kg) was administered to mice in the home cage environment. In the absence of cocaine memory reactivation, rapamycin had no effects on the established place preference; preference for the cocaine-paired compartment was maintained on days 11 and 18 (Figure 3B). These data indicate that mTORC1 activity is required for cocaine memory reconsolidation following memory reactivation.

### Experiment 3: Inhibition of p70S6K disrupted the reconsolidation of cocaine contextual memories

**FIGURE 4:**
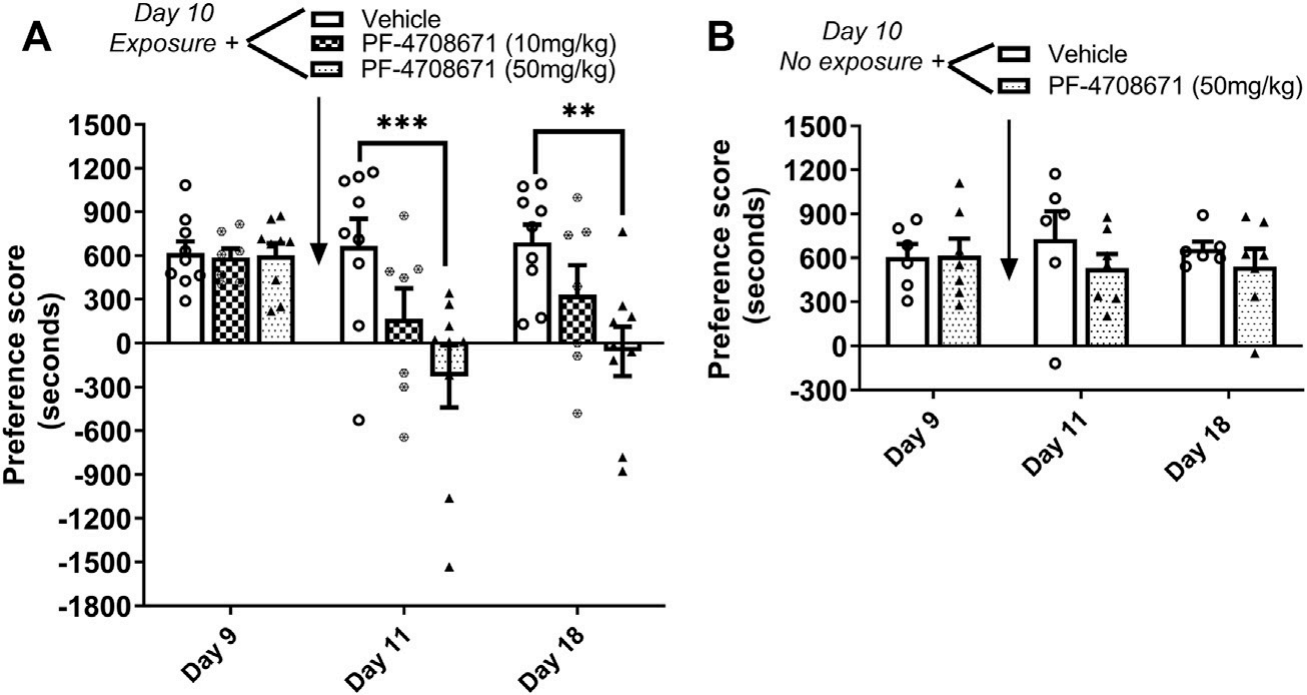
Inhibition of p70S6K attenuates reconsolidation of cocaine contextual memory. **(A)** Condition place preference was established with cocaine 10 mg/kg in three groups of mice as shown on day 9 during a 30-min test session. Mice were re-exposed to the cocaine-paired context in a drug-free state for 10 min to reactivate cocaine-associated memories on day 10. The p70S6K inhibitor, PF-4708671 (10 or 50 mg/kg ip), or vehicle was administered immediately after exposure to the cocaine compartment. Mice were re-tested for cocaine place preference 24 h and 7 d later. PF-4708671 (50 mg/kg) significantly abolished the previously established place preference when tested 24 h and 7 d after administration (days 11 and 18). Mice injected with vehicle maintained a significant cocaine place preference on days 11 and 18. **(B)** Condition place preference was established with cocaine 10 mg/kg in two groups of mice as shown on day 9. On day 10, mice remained in their home cages in the testing room and were injected with PF-4708671 (50 mg/kg ip) or vehicle. The test for cocaine place preference was repeated 24 h and 7 days later. PF-4708671 administered in the home cage (i.e., no exposure to the cocaine context) did not alter the previously established cocaine place preference; both vehicle and PF-4708671 groups maintained a cocaine place preference when retested on days 11 and 18 in the absence of re-exposure to the cocaine context. **p < 0.01, ***p < 0.001 for drug vs. vehicle at same time point; Data are expressed as means ± SEM. N = 9–10/group.

The role of p70S6 kinase in cocaine memory reconsolidation was investigated using a similar approach as Experiment 2. The p70S6K inhibitor, PF-4708671 (10 or 50 mg/kg ip), or vehicle was administered immediately following reactivation of cocaine memories or in the home cage on day 10. As shown in Figure 4A, repeated measures two-way ANOVA of preference scores revealed a significant interaction between the main effects of treatment and test day (interaction: F_4,44_ = 4.78, *p* < 0.01; PF-4708671 treatment: F_2,44_ = 5.05, *p* < 0.05; test day: F_2,44_ = 9.84, *p* < 0.001). Post hoc tests revealed that administration PF-4708671 (50 mg/kg) immediately following reactivation of cocaine reward memories significantly attenuated preference for the cocaine-paired chamber when tested 24 h later (*p* < 0.001 vs. vehicle, day 11) or 7 days later (*p* < 0.01 vs. vehicle, day 18). In the control study with no reactivation session on day 10, PF-4708671 (50 mg/kg) given in the home cage did affect the previously established cocaine place preference when tested 1 or 7 days later (Figure 4B). These results show that PF-4708671 was effective in abolishing an established cocaine place preference only when administered post memory reactivation, suggesting that p70S6 kinase is involved in reconsolidation of cocaine-contextual reward memories.

### Experiment 4: *Arc* mRNA expression was upregulated 60 and 120 min post reactivation of cocaine contextual memories

**FIGURE 5:**
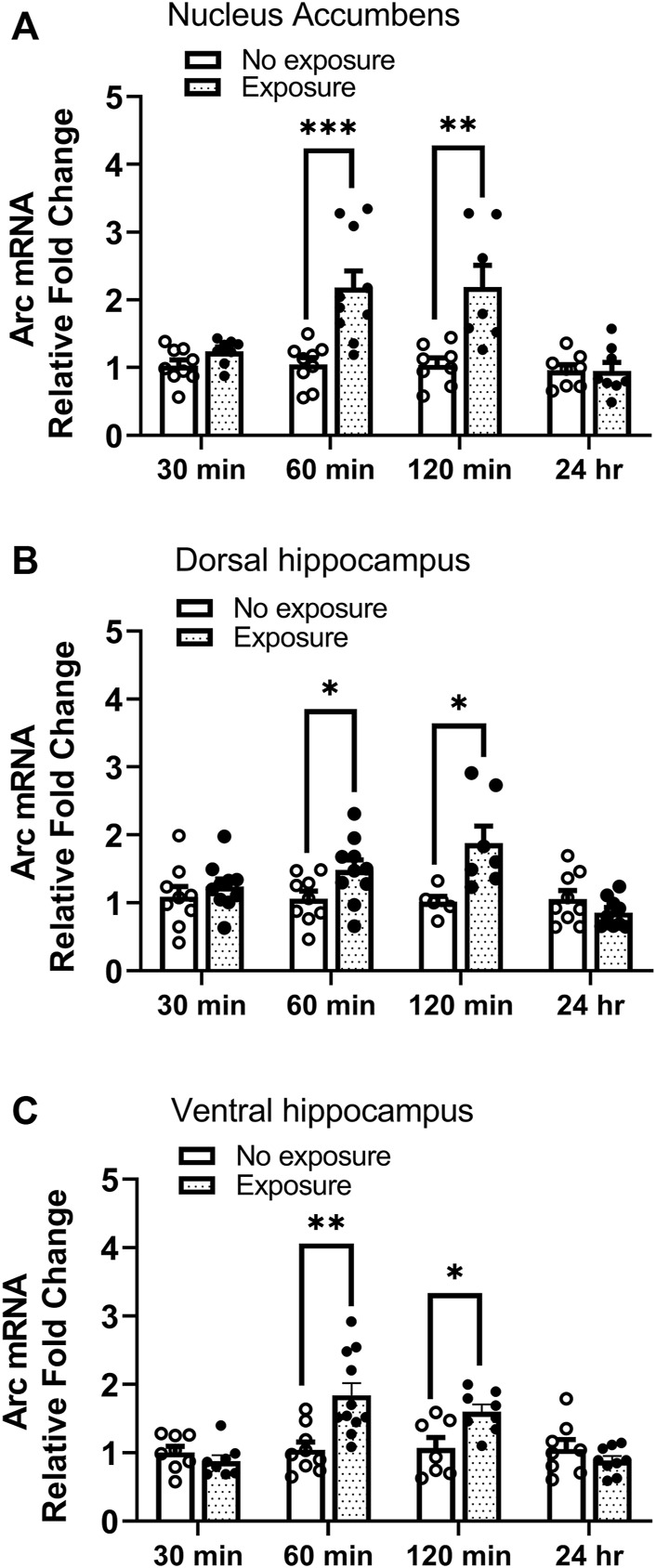
*Arc* mRNA is upregulated following reactivation of cocaine memories. Cocaine place preference was established with once daily conditioning sessions for 8 days, followed by a test for place preference on day 9. On day 10, 24 h after condition place preference was established, half the mice were re-exposed to the cocaine-paired compartment for 10 min while the other half remained in the home cage (i.e., no exposure). Brains were obtained 30 min, 60 min, 120 min, or 24 h later. Brain regions of interest were processed for qRT-PCR measurements of *Arc* mRNA expression. Levels of *Arc* mRNA were significantly elevated 60 and 120 min after exposure to the cocaine context in the **(A)** nucleus accumbens, **(B)** dorsal hippocampus, and **(C)** ventral hippocampus. No differences were noted at the 30 min or 24 h time points in any brain region. *p < 0.05, **p < 0.01, ***p < 0.001 for exposure vs. no exposure at same time point. Data are expressed as means ± SEM. N = 7–10/group.

*Arc* mRNA was measured 30 min, 60 min, 120 min, and 24 h following cocaine memory reactivation. As shown in Figure 5, *Arc* mRNA levels were significantly higher in nucleus accumbens (A. t = 4.04, df = 17, *p* < 0.001), dorsal hippocampus (B t = 2.197, df = 17, *p* < 0.05), and ventral hippocampus (C. t = 3.560, df = 18, *p* < 0.01) 60 min following the 10-min re-exposure to the cocaine context, as compared with expression in mice kept in their home cages (no exposure) without reactivation session. Moreover, the elevated *Arc* mRNA levels were maintained 2 h post reactivation in nucleus accumbens (A. t = 4.04, df = 17, *p* < 0.001), dorsal hippocampus (B. t = 2.197, df = 17, *p* < 0.05), and ventral hippocampus (C. t = 3.560, df = 18, *p* < 0.01). No changes in *Arc* expression were found 30 min or 24 h after memory reactivation (all *ps* > 0.05). These results show that *Arc* mRNA expression is upregulated following reactivation of cocaine contextual memories.

### Experiment 5: No changes in eIF4E-eIF4G interactions were found following reactivation of cocaine contextual memories

**FIGURE 6:**
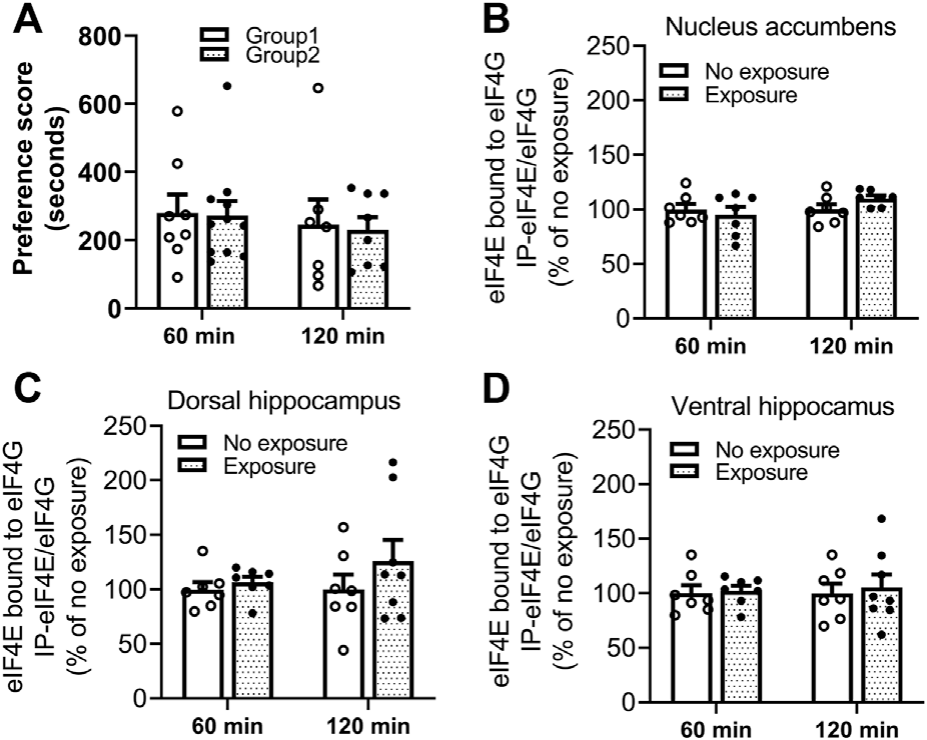
Reactivation of cocaine memories did not affect interactions between eIF4E and eIF4G. **(A)** Two groups of mice for each experimental time point (60 or 120 min) were conditioned with 10 mg/kg cocaine and saline once daily for 8 days. A 15-min post-conditioning test for cocaine place preference on day 9 showed similar preference for the cocaine-paired compartment. Cocaine memory was reactivated by exposure to the cocaine context for 10 min in a drug-free state, and brains obtained 60 or 120 min later. The eIF4E-eIF4G complex was measured by co-IP and Western blot methods. Levels of eIF4E bound to eIF4G in **(B)** nucleus accumbens, **(C)** dorsal hippocampus, and **(D)** ventral hippocampus were not significantly different in mice exposed to the cocaine context compared to the mice with no re-exposure. Data are expressed as % of no exposure controls, and means ± SEMs are shown. N = 7–10/group.

The interaction of eIF4E-eIF4G was measured by IP and western blot in tissues obtained 60 and 120 min following cocaine memory reactivation. As shown in Figure 6A, two groups of mice showed similar place preference scores on day 9. No significant differences in levels of eIF4E bound to eIF4G between exposure and no exposure groups of mice were found in nucleus accumbens (B. 60 min: t = 0.5521, df = 12, *p* > 0.05; 120 min: t = 1.864, df = 12, *p* > 0.05), dorsal hippocampus (C. 60 min: t = 0.7580, df = 12, *p* > 0.05; 120 min: t = 1.055, df = 13, *p* > 0.05) and ventral hippocampus (D. 60 min: t = 0.2381, df = 12, *p* > 0.05; 120 min: t = 0.3538, df = 13, *p* > 0.05) 60 or 120 min following the 10-min re-exposure to the cocaine-paired compartment. Representative immunoblots of nucleus accumbens, dorsal and ventral hippocampus from mice with or without exposure to the chamber previously paired with cocaine are included in the Supplementary Figures S3–S8. These data indicate that eIF4E-eIF4G interactions are not regulated in response to the reactivation of cocaine contextual memories in the nucleus accumbens or hippocampus at these time points.

## Discussion

The present findings demonstrate that mTORC1 activity is upregulated in the nucleus accumbens and hippocampus 60 min after cocaine memory reactivation as evidenced by increased phosphorylation of its target, p70S6K. Our previous study found that p70S6K phosphorylation was reduced immediately after cocaine memory reactivation (Shi et al., 2014), indicating the dynamic regulation of mTORC1 activity in nucleus accumbens and hippocampus following reactivation of cocaine memory. Inhibition of mTORC1 activity after cocaine memory reactivation abolished the previous established cocaine place preference, suggesting the requirement of mTORC1 in the reconsolidation of cocaine contextual memory. Moreover, p70S6K contributes to cocaine memory reconsolidation, as evidenced by attenuated cocaine place preference with systemic administration of a selective p70S6K inhibitor. Arc is a target gene of mTORC1 and its expression was upregulated in the nucleus accumbens and hippocampus following cocaine memory reactivation. The association of eIF4E-eIF4G was unchanged 60 and 120 min after reactivation of cocaine contextual memory.

Our previous report (Shi et al., 2014) suggests that reactivation of cocaine reward memory engages a signaling pathway consisting of Akt-GSK3β-mTORC1. GSK3β activity was induced following reactivation of cocaine memory and required for the reconsolidation of cocaine-associated memories. In contrast, mTORC1 activity, as assessed by phosphorylation of its target p70S6K, was downregulated in the hippocampus and nucleus accumbens immediately following cocaine memory reactivation (Shi et al., 2014). In the current study, the time-course of regulation of mTORC1 activity after cocaine memory reactivation was assessed through measurement of p70S6K phosphorylation (Hay and Sonenberg, 2004). mTORC1 activity was elevated in the hippocampus and nucleus accumbens 60 min after memory reactivation, which aligns with studies on reactivation of fear memories (Gafford et al., 2011, 2013; Jarome et al., 2018). This is the first report of the dynamic and bidirectional regulation of mTORC1 activity in response to reactivation of cocaine contextual memory.

Initial studies in the field supported the hypothesis that retrieval returns a consolidated memory to a labile state, which then requires a protein synthesis-dependent reconsolidation process to maintain the memory (Nader et al., 2000; Sara, 2000; Nader, 2003). However, accumulating evidence suggests that memory reconsolidation process is more complicated than initially thought. A growing body of literature suggest that both protein degradation and synthesis are required for reconsolidation of spatial and fear memory (Artinian et al., 2008; Lee et al., 2008; Jarome et al., 2011). For example, inhibition of protein degradation with the inhibitor lactacystin immediately, but not 3 h, after memory reactivation impairs the reconsolidation of spatial memory (Artinian et al., 2008). Several reports demonstrate that mTORC1 plays an important role in controlling a balance between protein synthesis and degradation during cell growth (Zhang et al., 2014; Zhao et al., 2015; Zhao J. et al., 2016; Boutouja et al., 2019). Inactivation of mTORC1 rapidly stimulates the ubiquitination and proteasomal degradation of many proteins in controlling cell growth (Zhao R. et al., 2016). Our finding of early reduction and later elevation of mTORC1 activity after reactivation of cocaine memory suggests that mTORC1 also may play an important role in controlling the balance between protein degradation and synthesis during reconsolidation of cocaine memory.

The current finding that inhibition of mTORC1 with rapamycin disrupted the previous established cocaine place preference suggests the requirement of mTORC1 in the reconsolidation of cocaine memory and supports a previous similar finding in rats (Lin et al., 2014). It is unlikely that rapamycin is enhancing extinction or causing extinction itself. The control groups showed no extinction upon retest on days 11 or 18, and there was no spontaneous recovery of place preference when tested 1 week post rapamycin administration. In a previous study (Lin et al., 2014), disruption of cocaine memory reconsolidation with rapamycin lasted at least 2 weeks, and a cocaine priming injection failed to reinstate cocaine-induced conditioned place preference. This is further support against rapamycin enhancing extinction; an extinguished memory will show spontaneous recovery or reinstatement in the presence of the unconditioned stimulus (i.e., drug priming). We further investigated the contribution of the mTORC1 downstream target p70S6K and found that inhibition of p70S6K with systemic administration of the specific inhibitor PF4708671 disrupted the reconsolidation of cocaine-associated memory. This indicates that p70S6K contributes to the essential role of mTORC1 in cocaine memory reconsolidation. In a prior investigation, PF4708671 did not affect reconsolidation of fear memories when measured 24 h later, but disrupted memory retention when measured 10 days after reactivation (Huynh et al., 2014). The discrepancies between their and our current findings may be due to the differences in type of memory trace (aversive fear memory vs. appetitive drug memory).

The interaction of eIF4E–eIF4G is regulated by 4E-BP1 and was investigated as a potential effector of mTORC1 involved in memory reconsolidation. No changes in eIF4E–eIF4G associations were found in the nucleus accumbens or hippocampus in response to reactivation of cocaine memory. This finding is similar to that of a previous study of fear memories which did not find changes in eIF4E-eIF4G interactions in the amygdala after reactivation of fear memories (Hoeffer et al., 2011). Although they found no alterations in eIF4E–eIF4G interactions in the amygdala following fear memory reactivation, Klann and colleagues went on to demonstrate that inhibition of eIF4E–eIF4G interactions with 4EGI-1 infused icv together with systemic administration of PF-4708671 diminishes fear memory measured either 24 h or 10 days after memory reactivation (Huynh et al., 2014), indicating the combined influence of eIF4E–eIF4G interactions and p70S6K1 activation in fear memory reconsolidation. Although the current results did not find alterations in eIF4E-eIF4G interactions following cocaine memory reactivation, the importance of this complex in process of reconsolidation cannot be ruled out. Further investigation at other time points after reactivation of cocaine memory and in other brain regions are necessary, in addition to functional studies of the complex to determine if reconsolidation of cocaine contextual memory is dependent on the eIF4E–eIF4G complex.

Prior work has shown that *Arc* expression is upregulated following exposure to contextual cues associated with drug (Schiltz et al., 2005) and cue-induced reinstatement of drug-seeking behavior after abstinence (Hearing et al., 2008a; Hearing et al., 2008b) or extinction (Koya et al., 2006; Zavala et al., 2008) using self-administration methods. Conditioned place preference is frequently used to investigate drug-context associated memories. Using this method, we found that *Arc* mRNA was higher in the nucleus accumbens and dorsal and ventral hippocampus 60 and 120 min after reactivation of cocaine contextual memory. Our findings are largely in agreement with a report that upregulation of Arc protein occurs following reactivation of cocaine-cue memory in rats, except no increase in Arc protein was found in the dorsal hippocampus (Alaghband et al., 2014). This discrepancy may be due to measurement of mRNA versus protein, different time points tested (60 or 120 vs. 40 min), or different species used in the two studies. There may be a delay between induced transcription (mRNA) and increased protein level (Liu et al., 2016) in the dorsal hippocampus. Other studies report disparities between levels of *Arc* mRNA and protein (Kelly and Deadwyler, 2003; McIntyre et al., 2005), which can be explained by the complexity of gene expression regulation under various scenarios (Liu et al., 2016). Interestingly, a previous report showed that *Arc* mRNA was highly induced 1–2 h after a single electroconvulsive seizure, and the levels of *Arc* mRNA return to near control levels about 6 h later, which is almost the same time window during which synaptic modifications are vulnerable to inhibition of protein synthesis (Steward and Worley, 2001). Arc protein is also elevated in the amygdala and hippocampus 90 min (Mamiya et al., 2009) or 2 h after retrieval of fear memory (Zhu et al., 2018). Given that *Arc* mRNA expression increased 60–120 min after retrieval of cocaine memory in the present study, *Arc* mRNA may be a marker of memory reconsolidation process. Arc expression can be regulated by mTORC1 (Takei et al., 2004; Barak et al., 2013). Evidence demonstrates that upregulation of Arc expression in response to alcohol memory reactivation is dependent on mTORC1 activity and can be abolished by inhibition of mTORC1 with rapamycin (Barak et al., 2013). Future studies will identify whether mTORC1 activity drives the enhanced *Arc* mRNA expression induced by cocaine memory reactivation.

The current study focused on regulation of mTORC1 and its potential downstream effectors in the mouse nucleus accumbens and hippocampus because of the known role of these regions in memory reconsolidation processes (Miller and Marshall, 2005; Milekic et al., 2006; Sakurai et al., 2007; Theberge et al., 2010; Noe et al., 2019). Results demonstrate regulation of mTORC1 activity and *Arc* expression in these regions following reactivation of cocaine contextual memories. The reconsolidation of cocaine memory was shown to be dependent on the activity of mTORC1 and p70S6K, although the site of action was not elucidated in this study. Several other brain regions have been implicated in the reconsolidation of drug reward memories including the basolateral amygdala (Milekic et al., 2006; Bernardi et al., 2009; Li et al., 2010; Theberge et al., 2010; Otis et al., 2013) and prefrontal cortex (Otis et al., 2013; Sorg et al., 2015). For example, inactivation of the basolateral amygdala with animycin after memory reactivation disrupts reconsolidation of morphine-conditioned place preference (Milekic et al., 2006) and β-adrenergic receptor blockade specifically in the basolateral amygdala impairs cocaine-associated memory reconsolidation (Otis et al., 2013). It is possible that multiple brain regions interconnect to regulate memory processes. Further studies are needed to elucidate the site of critical importance of mTORC1 signaling for the reconsolidation of cocaine contextual memory.

In summary, the data presented herein demonstrate that mTORC1 activity is necessary for reconsolidation of cocaine memory. p70S6K activity contributes to the essential role of mTORC1 in cocaine memory reconsolidation. Targeting drug memory reconsolidation processes could yield beneficial results in the prevention of cue-induced relapse to drug-seeking behaviors. Identification of the cellular processes that maintain drug-associated memory is important to achieve that goal.

## Data Availability

The raw data supporting the conclusions of this article will be made available by the authors, without undue reservation.
