# Supplementary material for: Reactivation of cocaine contextual memory engages mechanistic target of rapamycin/S6 kinase 1 signaling
Source: Front Pharmacol. 2022 Sep 27;13:976932. doi: 10.3389/fphar.2022.976932 (PMC9552424; doi:10.3389/fphar.2022.976932)
Supplement: Supplementary file 1 [file Table1.pdf]

1. **Experiment 1: Detection of phospho-p70S6K and total p70S6K in nucleus accumbens samples obtained 60 min after re-exposure to cocaine context**

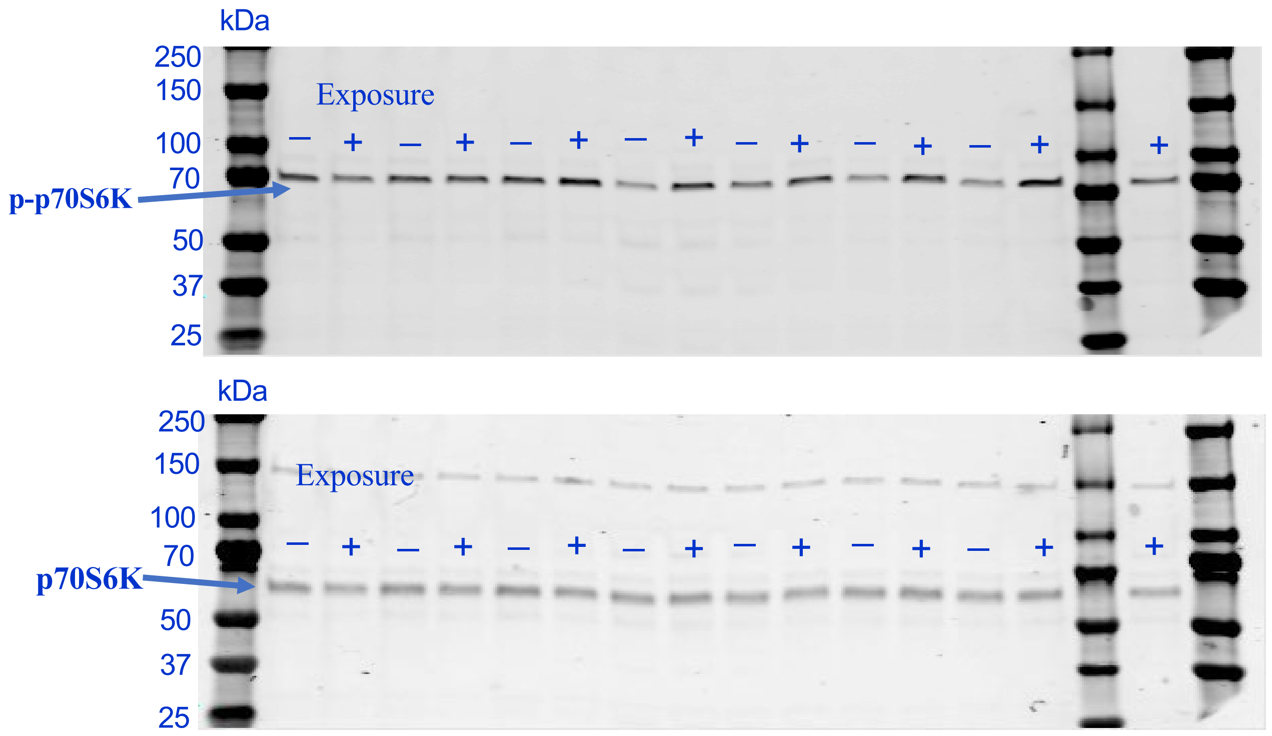

Figure S1: Immunoblots of phosphorylated-p70S6K (p-p70S6K; top) and p70S6K (bottom) in samples from the nucleus accumbens obtained 60 min after exposure to the environment previously paired with cocaine (Exposure +) or in home cage (Exposure -).

2. Experiment 1: Detection of phospho-p70S6K and total p70S6K in the hippocampus obtained 60 min after re-exposure to cocaine context

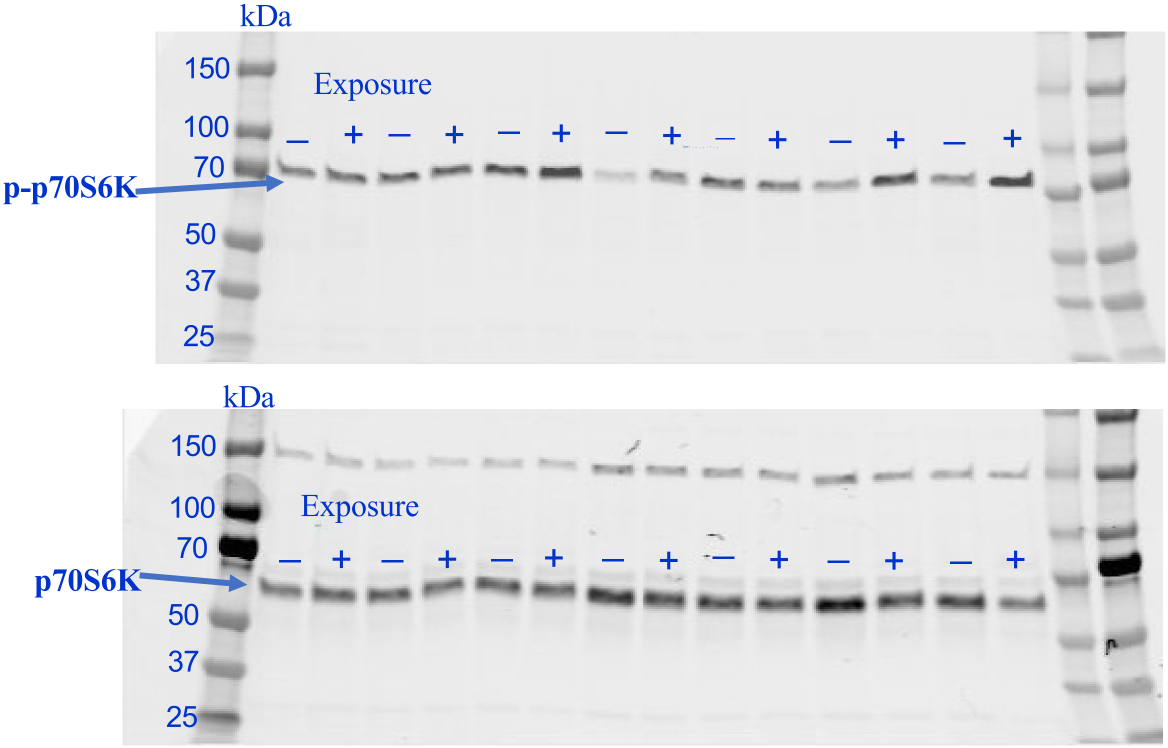

Figure S2: Immunoblots of phosphorylated-p70S6K (p-p70S6K; top) and p70S6K (bottom) in samples from the mouse hippocampus obtained 60 min after exposure to the environment previously paired with cocaine (Exposure +) or in home cage (Exposure -).

3. Experiment 5: eIF4E-eIF4G interactions in the nucleus accumbens 60 min after re-exposure to cocaine context

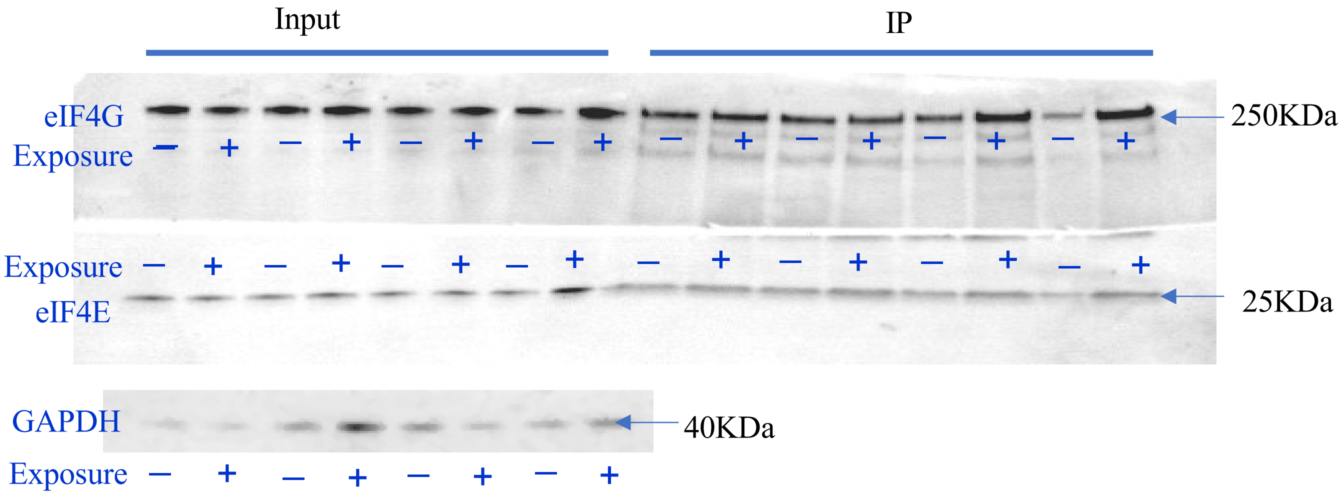

Figure S3: Immunoblots of eIF4G (top), eIF4E (middle) and GAPDH (bottom) in nucleus accumbens obtained 60 min after exposure to the environment previously paired with cocaine (Exposure +) or in home cage (Exposure -). Input: total tissue lysate; IP: immunoprecipitation

4. Experiment 5: eIF4E-eIF4G interactions in the dorsal hippocampus 60 min after re-exposure to cocaine context

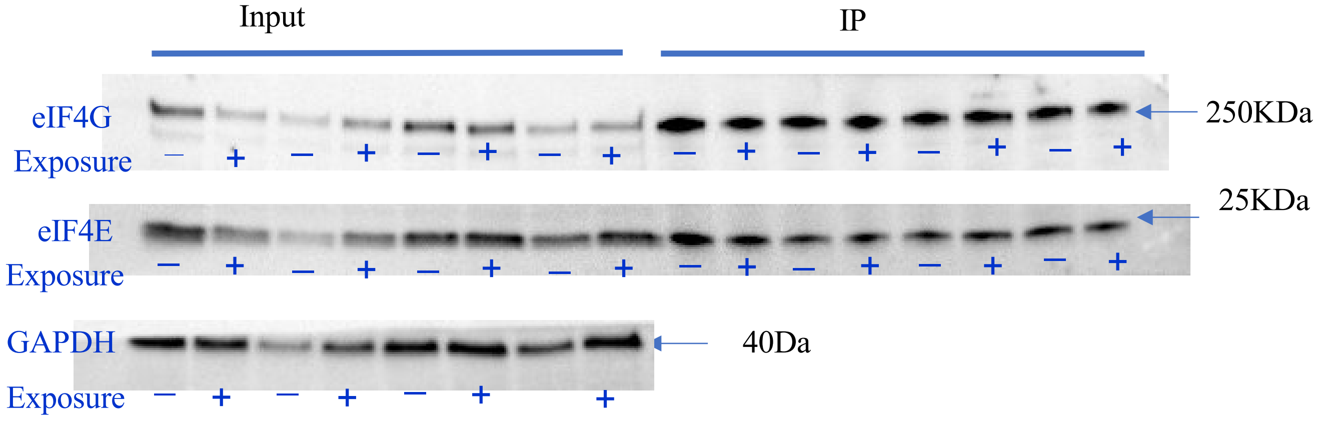

Figure S4: Immunoblots of eIF4G (top), eIF4E (middle) and GAPDH (bottom) in dorsal hippocampus obtained 60 min after exposure to the environment previously paired with cocaine (Exposure +) or in home cage (Exposure -). Input: total tissue lysate; IP: immunoprecipitation

5. Experiment 5: eIF4E-eIF4G interactions in the ventral hippocampus 60 mins after re-exposure to cocaine context

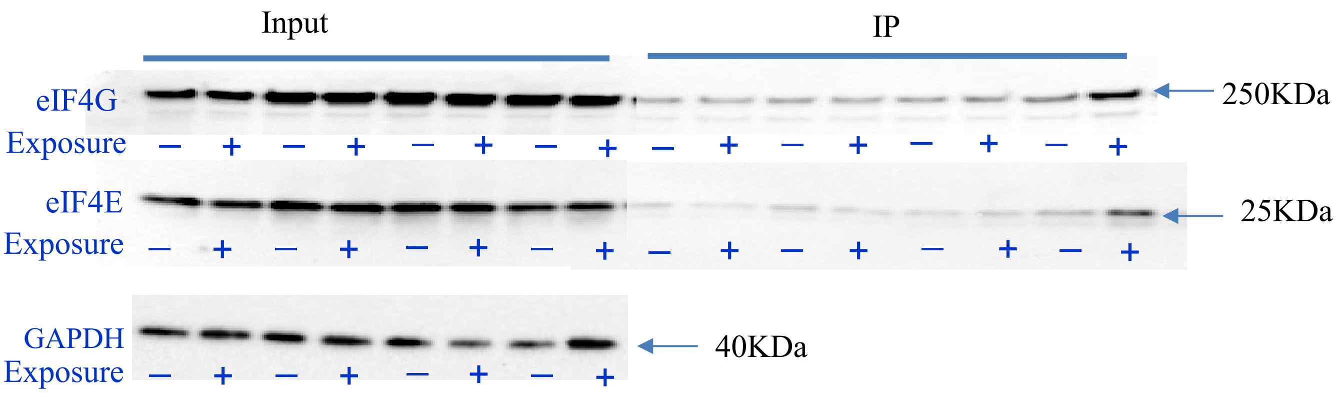

Figure S5: Immunoblots of eIF4G (top), eIF4E (middle) and GAPDH (bottom) in ventral hippocampus 60 min after exposure to the environment previously paired with cocaine (Exposure +) or in home cage (Exposure -). Input: total tissue lysate; IP: immunoprecipitation

6. Experiment 5: eIF4E-eIF4G interactions in the nucleus accumbens obtained 120 min after re-exposure to cocaine context

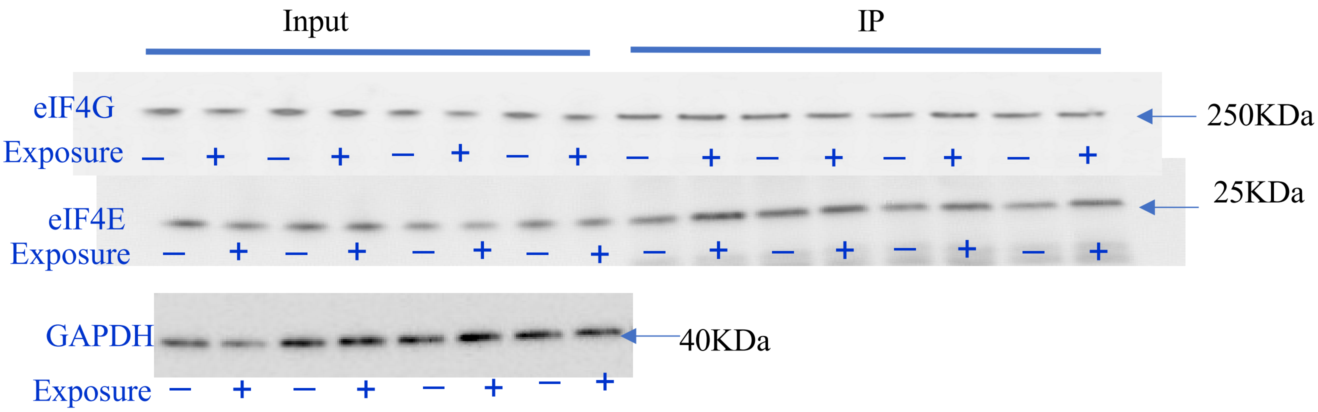

Figure S6: Immunoblots of eIF4G (top), eIF4E (middle) and GAPDH (bottom) in the mouse nucleus accumbens obtained 120 min after exposure to the environment previously paired with cocaine (Exposure +) or in home cage (Exposure -). Input: total tissue lysate; IP: immunoprecipitation

7. Experiment 5: eIF4E-eIF4G interactions in the dorsal hippocampus obtained 120 min after re-exposure to cocaine context

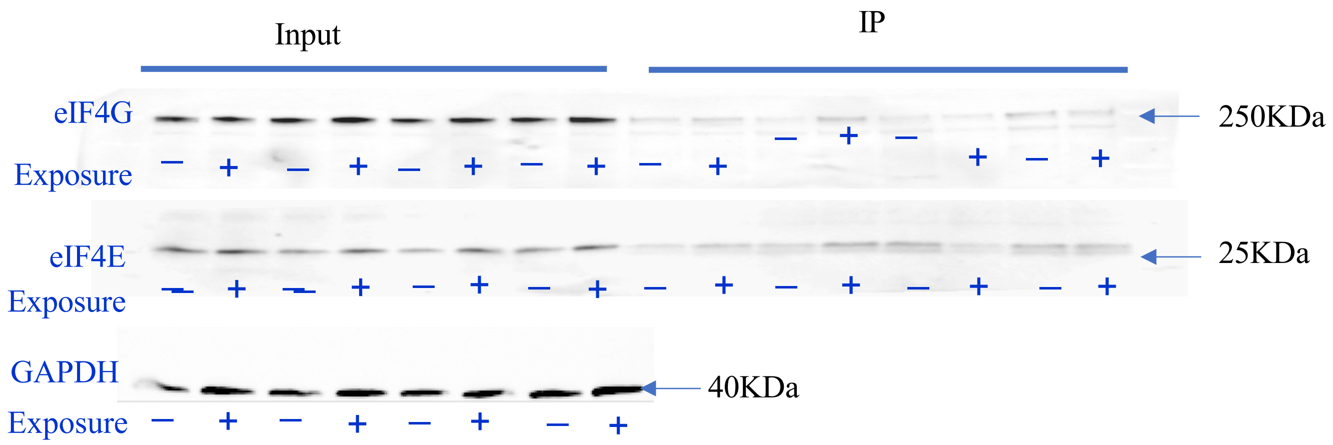

Figure S7: Immunoblots of eIF4G (top), eIF4E (middle) and GAPDH (bottom) in dorsal hippocampus obtained 120 min after exposure to the environment previously paired with cocaine (Exposure +) or in home cage (Exposure -). Input: total tissue lysate; IP: immunoprecipitation

8. Experiment 5: eIF4E-eIF4G interactions in the ventral hippocampus obtained 120 min after re-exposure to cocaine context

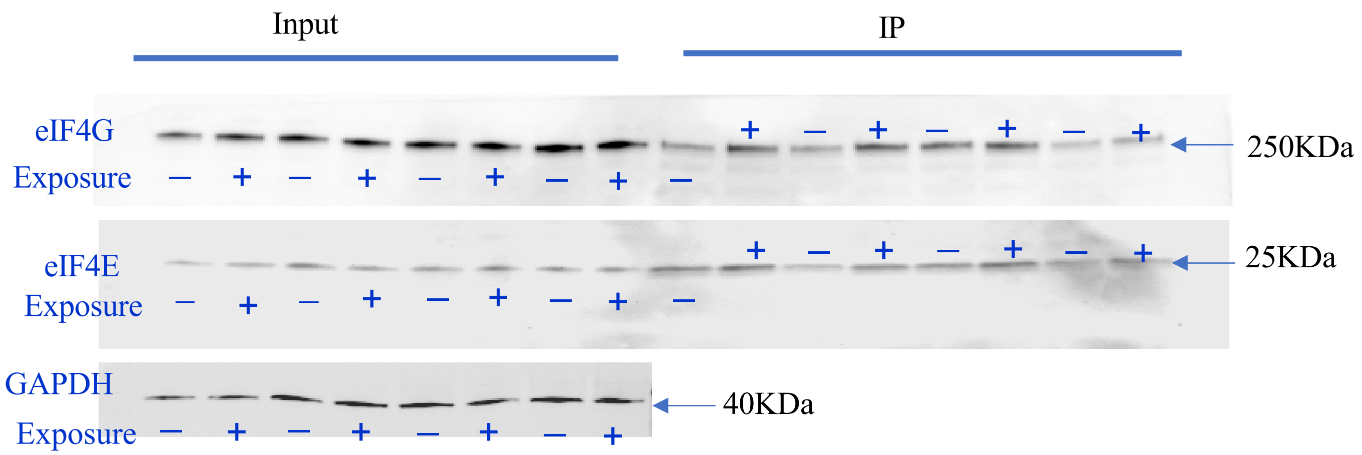

Figure S8: Immunoblots of eIF4G (top), eIF4E (middle) and GAPDH (bottom) in the ventral hippocampus obtained 120 min after exposure to the environment previously paired with cocaine (Exposure +) or in home cage (Exposure -). Input: total tissue lysate; IP: immunoprecipitation
